# How is circadian preference associated with cyber-victimization? A moderated mediation model of hostile recognition and online self-disclosure in Chinese early adolescent students

**DOI:** 10.3389/fpsyg.2022.970073

**Published:** 2022-11-15

**Authors:** Yanru Jia, Yuntena Wu, Tonglin Jin, Lu Zhang

**Affiliations:** School of Psychology, Inner Mongolia Normal University, Hohhot, China

**Keywords:** circadian preference, cyber-victimization, hostile recognition, online self-disclosure, eveningness

## Abstract

Although circadian preference is widely accepted to be a risk factor in the increase of adolescents’ negative experiences, little is known about its association with cyber-victimization. The current study sought to examine whether eveningness was significantly related to adolescents’ negative experiences. We further examined in-victimization events and whether hostile recognition and online self-disclosure played a vital role in eveningness and adolescents’ cyber-victimization. Study participants included 583 adolescents from four middle schools in China who completed questionnaires regarding their circadian preference, hostile recognition, online self-disclosure, and experience with cyber-victimization. Results indicated that adolescents with a high level of eveningness were more likely to experience cyber-victimization. Hostile recognition significantly mediated the relationship between eveningness and adolescents’ cyber-victimization. Furthermore, online self-disclosure moderated the indirect relationship between eveningness and cyber-victimization. Specifically, the paths from eveningness to hostile recognition and from hostile recognition to cyber-victimization became strengthened when adolescents experienced high levels of online self-disclosure. The results imply that researchers should pay more attention to remote factors, such as adolescents’ circadian preference and their relationship with cyber-victimization, to help them adapt to school requirements and reduce the frequency of victimization.

## Introduction

With the substantial intrusion of the Internet into teenage daily life, cyberbullying has become a significant problem that leads to adverse effects such as the internalization and externalization of psychopathology problems in adolescents (Chu et al., 2018). Especially as the recipient, the consequences borne by the victims are severe. Cyber-victimization is generally defined as situations when other individuals or groups repeatedly bully others using the Internet using various means including verbal intimidation, abuse, and malicious harassment (Vandebosch and Van Cleemput, 2008; Hu et al., 2014). Studies have shown that frequent and repeated cyber-victimization makes people vulnerable to prolonged low self-esteem and lack of social needs and that they demonstrate a sense of low value (Chen H. et al., 2020), increasing internalization problems such as negative emotions (Hu and Fan, 2013; Chu et al., 2018), and even suicidal ideation (Hinduja and Patchin, 2018). Therefore, there is an urgent need to better understand how to reduce the frequency of cyber-victimization.

Early adolescents are at particularly high risk of being bullied on the Internet. Indeed, the incidence of cyber-victimization in early adolescence is rather specific, with several studies showing that cyber-victimization peaks in junior middle school and declines in senior high school (Brochado et al., 2017; Morin et al., 2018). Early adolescence, as a stage in life, is crucial in the transition from immaturity to maturity. It is a period of a variety of psychological ups and downs when one is more sensitive to external threats and in a stage of heightened risk perception (Chandra-Mouli et al., 2018; Fontana et al., 2018). Early adolescents will also produce explosive internal and external bad performance during this period (Liao et al., 2021). Therefore, the current study chose to focus on early adolescents as the research object to explore the mechanism of cyber-victimization to understand why early adolescents face a greater risk of cyber-victimization.

## Literature review and hypothesis development

### Eveningness and adolescents’ cyber-victimization

Circadian preference refers to an individual’s time preference for daily activities and sleep habits, and is based on the circadian rhythm of sleep-awakening-activity over the course of 24 h (Au and Reece, 2017; Chen Y. J. et al., 2020). Regarding circadian preference, each of us is in such a continuum, and the ends of the continuum are often vividly called “larks” (morningness) and “owls” (eveningness). Moreover, most of us fall between these two categories (Natale and Cicogna, 2002). Among them, the evening-type prefers to go to bed and rise late, feeling and performing better in the afternoon or evening (Fredrick et al., 2022). Although it should be avoided to associate eveningness preference with some negative things simply, most studies suggest that eveningness is often regarded as a potential risk factor for adolescent health problems (Dolsen and Harvey, 2017; Chen Y. J. et al., 2020). This high risk may be due to persistent social jet lag in this particular group (Komada et al., 2016). For early adolescents, night-owls like to stay up late and sleep in, but due to their everyday studies, they generally must arrive at school around 7:00–8:00 in the morning, which causes them to wake up at a time that is out of sync with their biological clock (Adan et al., 2012; Tonetti et al., 2015). Experiencing the mismatch between sleep time, best performance, and early morning class time (Dolsen et al., 2019), teenagers with eveningness preference will accumulate sleep debt over the week, resulting in adverse “social jet lag” and a series of other adverse consequences, such as lack of sleep, excessive stress, and social conflict (Roenneberg et al., 2012; Díaz-Morales et al., 2014; Chung et al., 2018).

So, does eveningness preference also lead to an increased threat of cyber-victimization? Thus far, no research has explored the relationship between the two. The dual-regulation process model of sleep may help explain the effect of evening-type on adolescents’ cyber-victimization. This hypothesis proposes that an individual’s sleep-awakening cycle is regulated by rhythmic and homeostasis processes (that is, sleep drive). These two systems work together to promote stable sleep rhythms and high-quality sleep security in individuals (Dijk and Lockley, 2002). However, social rhythms interfere with the synchronization of biological rhythms, thus affecting cognitive and behavioral changes (Adan et al., 2012). For early adolescents with evening-type preference, the internal biological clock of “getting up late” conflicts with the external social time of “getting up early,” making it impossible for them to carry out their scheduled activities during a stable sleep-awakening time (Song and Zheng, 2014). As a result, these adolescents are in an unstable state of stress defense and are more likely to perceive threatening clues in the social environment and react more strongly. This is further reinforced by the absence of experience in the online environment, resulting in more judgment around being attacked leading to them reporting a higher level of cyber-victimization. Empirical studies have also found that the more likely circadian preference tends to eveningness, the more apparent adolescents’ emotional responses, stress arousal, and stress answers will be (Jankowski and Ciarkowska, 2008; Jankowski et al., 2014). Therefore, we can infer that people with an eveningness preference will suffer a higher level of cyber-victimization.

Although there is some evidence showing that eveningness plays an important role in cyber-victimization, less is known about its potential mechanism: How is it that adolescents with evening-type preference experience more cyber-victimization? Does eveningness affect cyber-victimization by changing an individual’s cognitive style (e.g., hostile recognition), and what is the boundary condition (e.g., depth and breadth of online disclosure) between these two?

### The mediating role of hostile recognition

Considering the way that information is transmitted in online social communication, hostile recognition may be an important explanatory variable for the relationship between evening-type adolescents and cyber-victimization. Hostile recognition refers to the automatic and long-term hostile thoughts and hostile interpretation bias the individuals form in communicating with others, evidenced by revenge, negation, and negative evaluation (Snyder et al., 1997). Electronic communication in online social communication is particularly unique as it is a perceptual consciousness activity that lacks factual information (e.g., facial expression). Its effectiveness depends on the perceptual cue processing of the information receiver, that is, the information receiver must interpret and explain the meanings of online information subjectively and objectively by imbuing online text information with additional subjective emotions (Kowalski et al., 2014; Liu, 2019). Therefore, in the case of cyber-victimization, whether another’s verbal or written information is interpreted as hostile is an essential factor affecting individuals’ judgment of whether they are being bullied online.

From a physiological point of view, eveningness may be connected with more hostile thinking. Studies have shown that evening-type adolescents have higher neurotic scores, which are associated with more anxiety, hostility, impulsiveness, and vulnerability (Gaspar-Barba et al., 2009; Tonetti et al., 2009). Meanwhile, according to the comprehensive cognitive model, a positive effect of hostile recognition is that it affects one’s initial stage of processing and interpretation of social situations. In hostile recognition, individuals tend to understand current social situational cues as being hostile (Wilkowski and Robinson, 2008), such as identifying inoffensive language errors as threats they use to create more complex judgments of victimization, thus reporting higher levels of cyber victimization. Empirical research has also found that eveningness can prejudice individuals to regard social information as negative, improve individual punishment sensitivity, and indirectly affect subsequent social responses (Markarian et al., 2019). Thus, it is reasonable to expect that eveningness could significantly increase adolescents’ cyber-victimization. Therefore, it can be reasonably assumed that eveningness indirectly affects early adolescents’ cyber-victimization by changing their level of hostile recognition.

### The moderating role of online self-disclosure

Online self-disclosure refers to behavior by which individuals use a variety of ways to transmit information to others to maintain online communication or satisfy their personal needs and can be divided into depth and breadth of disclosure, that is, the privacy and scope of online self-disclosure (Xie et al., 2013). Previous studies have focused on the positive role of online self-disclosure. For example, the process model of self-disclosure suggests that individuals seek social support through self-disclosure to improve their mental health (Chaudoir and Fisher, 2010). However, online self-disclosure also has adverse effects, that is, it can increase the frequency and severity of harmful events.

According to the victim-driven model, the content or means of self-disclosure (e.g., unintentional or uninformed behavior) in online social spaces may lead to negative emotions regarding potential bullies, which in turn can increase the possibility of cyber-victimization (Grubb and Turner, 2012). In terms of the breadth and depth of online self-disclosure, a high level of online self-disclosure increases the possibility that eveningness students will be exposed to potential bullies and become bullied online. In contrast, a low level of online self-disclosure may play a buffer role and help students avoid cyberbullying. Research has also shown that young adolescents sharing personal information on the Internet leads to threatened personal privacy and online security (Zimmer et al., 2010), which also leads to a greater likelihood of cyber-victimization (Bryce and Klang, 2009). Thus, it is reasonable to propose that online self-disclosure will moderate the relationship between eveningness and cyber-victimization in early adolescents.

### The current study

The present study aimed to explore why early adolescents are at greater risk of cyber-victimization. Specifically, we tested whether eveningness predicts cyber-victimization in a large sample of 583 Chinese adolescents, ranging from 12 to 16 years of age. Moreover, we explored the mechanism of the relationship, that is, the mediating role of hostile recognition and the moderating role of online self-disclosure. The intent was to further theoretical guidance and intervention suggestions to prevent and treat cyber-victimization of early adolescents.

With consideration of the aforementioned research findings, then, we proposed the following hypotheses:

Hypothesis 1: Eveningness positively predicts early adolescents’ cyber-victimization.

Hypothesis 2: Hostile recognition mediates the association between eveningness and cyber-victimization.

**FIGURE 1:**
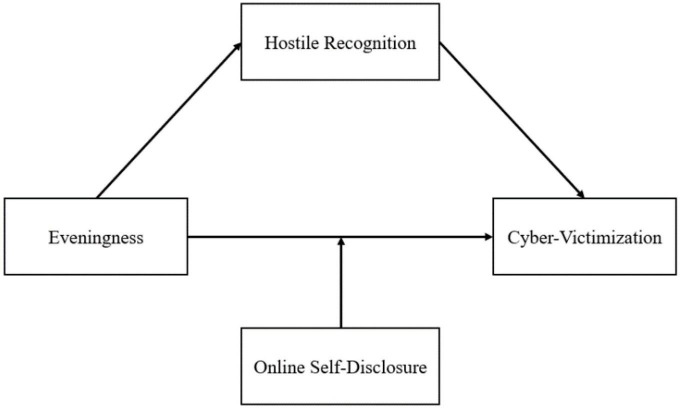
Conceptual model.

Hypothesis 3: Online self-disclosure moderates the relationship between eveningness and cyber-victimization (see Figure 1).

## Materials and methods

### Participants

A total of 600 middle school students were selected by random cluster sampling from four Chinese middle schools. In total, 583 valid responses were collected (male = 253; female = 330). The participants were representative of the overall sample in terms of age, gender, grade, and place of origin (i.e., whether the participants came from rural or urban areas). The participants ranged from 12 to 16 years of age (Mage = 13.90, SD = 1.71), and were in first, second, and third grades, with a sample size of 195 (33.4%), 231 (39.6%), and 157 (26.9%), respectively. All subjects completed the questionnaires during class time, which focused on eveningness, cyber-victimization, hostile recognition, and online self-disclosure. The study obtained the informed consent of all participants as well as the approval of the local ethics committee before commencing.

### Measures

#### Eveningness

Eveningness was assessed using the Morningness-Eveningness Stability Scale (MESSi, Randler et al., 2016). The 15-item MESSi consists of three dimensions that describe one’s circadian preference and circadian fluctuations. The eveningness dimension comprises five items (e.g., “I am usually in an excellent mood in the evening”), all of which are rated on a 5-point scale. Higher scores represent a higher degree of eveningness. CFA indicated that eveningness showed acceptable data fit: χ*^2^/df* = 1.75, CFI = 0.99, TLI = 0.99, RMSEA = 0.04, and SRMR = 0.02. Cronbach’s α for the scale in the current study was 0.71.

#### Cyber-victimization

Cyber-victimization was assessed using the Cyberbullying Inventory (CBI, Erdur-Baker and Kavşut, 2007). The 18-item CBI consists of one dimension that describes one’s frequency of cyber-victimization (e.g., “I’ve been hurt by people I know online”). This study used the Chinese version of the CBI (Zhou et al., 2013), in which items are rated on a 5-point scale (1 = “never encountered” and 4 = “more than five times”). Higher scores represent a higher frequency of experiencing cyber-victimization. CFA indicated that cyber-victimization showed an acceptable data fit: χ*^2^/df* = 4.91, CFI = 0.92, TLI = 0.91, RMSEA = 0.08, and SRMR = 0.02. Cronbach’s α for the scale in the current study was 0.94.

#### Hostile recognition

Hostile recognition was measured using the Buss-Perry Aggression Questionnaire (BPAQ, Buss and Perry, 1992). The 22-item BPAQ consists of four dimensions that describe the level of one’s aggression. The hostile recognition dimension contains eight items (e.g., “Sometimes I feel like people are laughing at me behind my back”), each of which is rated on a 5-point scale. This study used the Chinese version of the BPAQ (Lv et al., 2013). CFA indicated that the hostile recognition dimension showed an acceptable data fit: χ*^2^/df* = 4.22, CFI = 0.98, TLI = 0.97, RMSEA = 0.07, and SRMR = 0.04. Cronbach’s α for the scale in this study was 0.93.

#### Online self-disclosure

Online self-disclosure was assessed using the Self-Disclosure Index Scale (SDIS, Miller et al., 1983). The 10-item SDIS consists of one dimension that describes the depth and breadth of the information one is exposed to *via* online networks (e.g., “There are things I have done which I feel guilty about”). The current study used the Chinese version of the SDIS (Li et al., 2006), in which items are rated on a 5-point scale (1 = “Never tell each other” and 5 = “Tell each other in great detail”). A higher score represents a higher degree of online self-disclosure. The original scale asks respondents to consider four types of target audiences when answering about their degree of online self-disclosure. In the current study, however, referring to existing research (Xie et al., 2016), the target audience was changed to be only the respondents’ social media friends, examining the respondents’ self-disclosure to these friends through social media. CFA indicated that the SDIS showed an acceptable data fit: χ*^2^/df* = 4.63, CFI = 0.97, TLI = 0.94, RMSEA = 0.08, and SRMR = 0.08. Cronbach’s α in the current study for this scale was 0.70.

#### Analytical strategies

Data were collected through online surveys. Participants were assured that their answers were confidential and anonymous and would only be used for academic research. Before responding to the actual questions for cyber-victimization, participants were asked to read through the background description of cyberbullying carefully and to respond to a single item first, replying either “I see” or “I will read it again.”

When exploring the data, we first analyzed the scores of the questionnaires through descriptive statistics and correlations using SPSS 24.0. We then tested the moderated mediation effect using Mplus 7.4 to provide bootstrap confidence intervals (CI) as part of the data processing. Covariates were controlled in both the mediation and moderated analyses.

## Results

### Common method bias test

As the data collected in this study were all self-reported, a common method bias test was necessary before continuing with follow-up analysis and processing. Unrotated exploratory factor analysis of the measurement items of all variables found that six common factors with eigenvalues greater than 1 were proposed, and the first common factor explained 33.06% of the total variation and less than 40% of the total variation (Zhou and Long, 2004). Therefore, no serious problem of common method bias was found in the current study.

### Descriptive and Pearson’s correlation results

The obtained associations among variables were largely consistent with past findings (see Table 1). Eveningness was positively related to both cyber-victimization (*r* = 0.16, *p* < 0.01) and hostile recognition (*r* = 0.17, *p* < 0.01). Cyber-victimization was positively related to both hostile recognition (*r* = 0.32, *p* < 0.01) and online self-disclosure (*r* = 0.22, *p* < 0.01). Finally, hostile recognition was positively related to online self-disclosure (*r* = 0.24, *p* < 0.01).

**TABLE 1:** Descriptive statistics and correlation matrix among principal variables (*n* = 583).

Variables	*$\bar{x}$* ± s	1	2	3	4
1. Eveningness	3.40 ± 0.74	1			
2. Cyber-victimization	1.29 ± 0.46	0.16**	1		
3. Hostile recognition	2.78 ± 0.88	0.17**	0.32**	1	
4. Online self-disclosure	2.75 ± 0.48	0.08	0.22**	0.24**	1

**p < 0.01.

### Moderated mediation effect analysis

**FIGURE 2:**
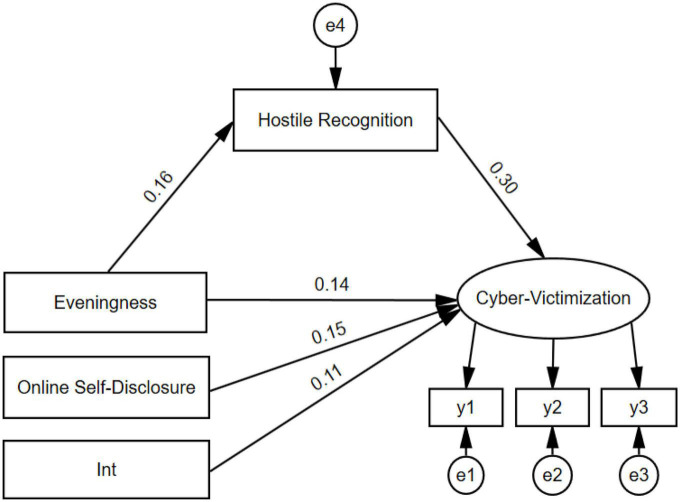
Moderated mediation model. **(A)** Int is the interaction term about online self-disclosure and eveningness. **(B)** y1, y2, and y3 are three indicators for the item parceling of cyber-victimization.

To test the theoretical hypotheses, a structural equation model was constructed to test and analyze the influence mechanism of eveningness on early adolescents’ cyber-victimization. The results showed that the overall fitting result of the model was good (χ*^2^/df* = 4.09, CFI = 0.97, TLI = 0.94, SRMR = 0.05, RMSEA = 0.07; see Figure 2). Eveningness was shown to significantly predict cyber-victimization of early adolescents (β = 0.14, *t* = 4.37, *p* < 0.01), and had a significant predictive effect on hostile recognition (β = 0.16, *t* = 3.60, *p* < 0.01). Hostility recognition had a significant effect on the prediction of cyber-victimization (β = 0.30, *t* = 7.62, *p* < 0.01), with a 95% CI of [0.07, 0.25]. Eveningness had an indirect effect on cyber-victimization through hostile recognition, with a 95% CI of [0.05, 0.02], that is, eveningness was shown to indirectly affect the level of cyber-victimization by affecting early adolescents’ hostile recognition. Furthermore, the interaction between online self-disclosure and eveningness had a significant predictive effect on cyber-victimization (β = 0.11, *t* = 3.00, *p* < 0.01) with a 95% CI of [0.03, 0.17], that is, the results confirmed the regulatory effect of online self-disclosure.

**FIGURE 3:**
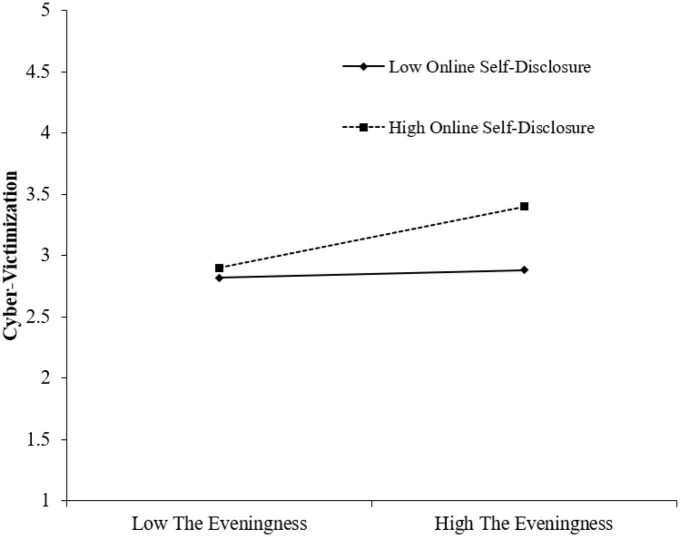
Online self-disclosure as moderator on the relationship between eveningness and cyber-victimization.

To investigate the utility level of each variable at different levels, the current study created high and low groupings of online self-disclosure (*M*± *1SD*). Based on the overall model, we investigated the regulatory effect of online self-disclosure and found that, at the low level of online self-disclosure (*M*-*1SD*), eveningness had a lower level of prediction of early adolescents’ cyber-victimization (β = 0.01, *p* > 0.05), with a CI of [–0.18, 0.14], which included 0. Meanwhile, at the high level of online self-disclosure (*M* ± *1SD*), eveningness had a higher level of prediction of cyber-victimization (β = 0.33, *p* < 0.01), with a 95% CI of [0.30, 0.93], which excluded 0. Overall, lowering of one’s level of online self-disclosure enhanced the predictive effect of eveningness on cyber-victimization (see Figure 3).

## Discussion

### Eveningness and adolescents’ cyber-victimization

The results showed that eveningness in early adolescents had a significant positive predictive effect on cyber-victimization, which verifies Hypothesis 1. In online environments, early adolescents with an evening-type circadian preference of getting up and going to bed late are shown to have a higher risk of cyber-victimization. To put it simply, eveningness is a dangerous condition for the circumstance of early adolescent cyber-victimization. According to the 2020 China Sleep Index Report, 58.9% of those questioned reported staying up late more than three times a week on average, of which 49.4% chose to stay up late voluntarily; moreover, of those staying up late actively, the majority were the generation after 00s, 95s, and 90s (China Sleep Research Society, 2021). The diversity of entertainment activities available in the online environment may cause the shift in sleep patterns of those early adolescents born in 2005 and later, creating more conflict conditions between their internal biological clocks and external social schedules and demands. With this background of an increased rate of evening-type preference among early adolescents, they become more alert to social threat cues, which then strengthens their observation of the details of their physical environment, causing them to be more sensitive to subtle hostile comments leading them to make more cyber-victimization judgments and self-reports when faced with ambiguous online situations (Somerville, 2013; Alipan et al., 2020).

### The mediating role of hostile recognition

In addition to testing the direct effect between eveningness and cyber-victimization, this study also examined the mechanism between them. This was done in two parts, by looking at both the mediating role of hostile recognition and the moderating effect of online self-disclosure.

The results of this study support the mediating effect of hostile recognition between eveningness and cyber-victimization of early adolescents, which validates Hypothesis 2. On the one hand, eveningness positively predicted hostile recognition in early adolescents, which supports the results of other studies. Eveningness groups are more likely to adopt negative or evasive ways to solve problems when they encounter difficulties and setbacks, which in turn leads to a surge of negative emotions (Antúnez et al., 2015). Watts and Norbury (2017) found that individuals with an evening-type preference have poorer cognitive reevaluation ability and stronger expression inhibition ability than those with a morning-type preference, and this abnormal emotion regulation ability can further affect their level of hostile recognition. According to cognitive resource theory, adolescents with an evening-type preference will be deprived of sleep by having to engage in learning activities such as “morning reading” sessions as required by their schools, causing them to consume more cognitive resources to help them adapt to the morning-focused biological clock that wider society generally follows. This then leads them to perceiving events as being more unpleasant and use more negative explanations to interpret situations. In the long run, these adolescents form a poor cognitive style of hostile cognition.

On the other hand, one’s level of hostile recognition can significantly predict early adolescents’ cyber-victimization, which supports the comprehensive cognitive model, that is, the automatic extraction of cues and hostile interpretation will lead to an increase in early adolescents’ perceptions of instances of cyber-victimization. This partly explains why one of the issues in the process of prevention and treatment of cyber-victimization behavior is that it is difficult to evaluate. Whether one is being bullied or is a bystander, it is often difficult to tell whether others’ words and deeds are well-intentioned jokes or malicious bullying. If general online behavior is mistakenly judged as cyber-victimization in ambiguous situations, this can lead to increased conflict and cause event participants more harm (Alipan et al., 2020). Hostile cognition aggravates this negative assessment further, causing individuals to tend to choose to interpret a situation as “malicious bullying” rather than simply a “well-intentioned joke” when they are faced with suspected cyber-victimization and misinterpreting positive information from others and judging online social behaviors as bullying—whether it is or not (Markarian et al., 2019). In the case of early adolescents, the current study findings showed that eveningness led to increased negative hostility which affected their interpretation of cyber-victimization and caused adolescents to report more incidences of cyber-victimization, which may include some misjudgments or overreactions.

### The moderating role of online self-disclosure

The results of this study support the regulatory role of online self-disclosure in the influence of the pathway of eveningness on early adolescents’ cyber-victimization. Furthermore, they showed that online self-disclosure can significantly promote adolescents’ perceived cyber-victimization. Specifically, when one had a higher level of online self-disclosure, eveningness had a significant positive predictive effect on early adolescents’ cyber-victimization, while at a lower level of online self-disclosure, the predictive effect was not significant. This result confirms Hypothesis 3 of the current study. For adolescents with a high level of online self-disclosure, it may be easier to reveal more in the wide open online environments, but individuals are not able to control the consequences of their disclosures. They may experience negative consequences or even become the target of prejudices, while, simultaneously, the more one reveals, the more vulnerable they are to external attacks. However, the first occurrence of cyber-victimization must meet the condition of participation. For adolescents with a low level of online self-disclosure, their reduced frequency of online participation and, indeed, their lower level of online self-disclosure itself may help them protect themselves better in online environments. Therefore, online self-disclosure may be the boundary condition for “eveningness can predict early adolescents’ cyber-victimization.” Correctly controlling the amount, depth, and breadth of online self-disclosure can benefit adolescents in their engagement in social interactions online.

### Education and intervention

Existing anti-bullying interventions tend to focus more attention on cyberbullies, bystanders, or social environment perspectives, despite the effect of these interventions being relatively weak (Nocentini et al., 2018). Through the results of this study, however, we can begin to shift intervention attention to the risk factors that more directly affect cyber-victimization. Through improved interventions, some of which we propose below, we can reduce adolescents’ perception of cyber-victimization to better prevent and mitigate incidences of cyber-victimization.

Considering the difficulty and cost of intervention, we should first pay attention to those more malleable factors, such as the regulatory variable selected in this article—online self-disclosure. Online self-disclosure has already been shown to be a double-edged sword (Chaudoir and Fisher, 2010). Educators can guide teenagers in how to make more appropriate online self-disclosures through classroom teaching or other settings and the means to reduce adolescents’ exposure to cyber-victimization incidents in hopes of reducing the frequency of cyber-victimization. Just as the old saying goes, “Master’s words and actions move heaven and earth; may he be careless in regard to them?,” understanding the impacts of various depths and breadths of self-disclosure can lead to adolescents making wiser choices and better understanding that caution in words and deeds is an effective and important method of self-protection in the online world. Second, we can work to correct the detrimental cognitive style of hostile cognition through cognitive training or neural stimulation, to improve early adolescents’ abilities to detangle attention from negative information (Tonta et al., 2019). Finally, as circadian preference is related chiefly to genes, personality, social jet lag, and other factors that are difficult to intervene with, the effect of the intervention will be difficult and slow. But biological clock therapy can also be used to intervene in adolescents’ circadian preferences. In terms of physical means, one can increase their level of proper exercise or increase their daylight time. In terms of psychological means, skills such as mindfulness training can help evening-type adolescents shift their sleep rhythms and reverse their experience of bad social jet lag (Chen Y. J. et al., 2020).

## Data availability statement

The original contributions presented in this study are included in the article/supplementary material, further inquiries can be directed to the corresponding author.

## Ethics statement

The studies involving human participants were reviewed and approved by the School of Psychology, Inner Mongolia Normal University. Written informed consent to participate in this study was provided by the participants’ legal guardian/next of kin.

## Author contributions

YJ, TJ, and LZ contributed to the experiment design, investigation analysis of the data, and drafted the manuscript. YW helped to perform the revision of the manuscript and provided final approval for the manuscript. All authors contributed to the article and approved the submitted version.
